# Simultaneous Extensive Intraductal Papillary Neoplasm of the Bile Duct and Pancreas: A Very Rare Entity

**DOI:** 10.1155/2016/1518707

**Published:** 2016-01-26

**Authors:** Vor Luvira, Ake Pugkhem, Theerawee Tipwaratorn, Yaovalux Chamgramol, Chawalit Pairojkul, Vajarabhongsa Bhudhisawasdi

**Affiliations:** ^1^Department of Surgery, Faculty of Medicine, Khon Kaen University, Khon Kaen 40002, Thailand; ^2^Department of Pathology, Faculty of Medicine, Khon Kaen University, Khon Kaen 40002, Thailand

## Abstract

Intraductal papillary neoplasm of the bile duct (IPNB) is a specific type of bile duct tumor. It has been proposed that it could be the biliary counterpart of the intraductal papillary neoplasm of the pancreas (IPMN-P). This hypothesis is supported by the presence of simultaneous intraductal tumors of both the bile duct and pancreas. There have been five reports of patients with simultaneous IPNB and IPMN-P. In all of these cases, biliary involvement was limited to the intrahepatic and perihilar bile duct, which had characteristics similar to IPMN-P and usually had slow progression in nature. Herein, we present the first case of extensive intraductal neoplasm involving the extrahepatic bile duct, intrahepatic bile duct, and entire length of the pancreas with a poor outcome, even after being treated aggressively with radical surgery and adjuvant chemotherapy. Additionally, we summarize previous case reports of simultaneous intraductal lesions of the bile duct and pancreas.

## 1. Introduction

Intraductal papillary neoplasm of the bile duct (IPNB) is a specific type of bile duct tumor. There are differences in the natural histories of IPNB and conventional cholangiocarcinoma. IPNB develops through the adenoma-carcinoma sequence and usually progresses slowly [1]. Recent studies have shown similarities between intraductal papillary neoplasm of the bile duct and intraductal papillary mucinous neoplasm of the pancreas (IPMN-P) [2]. It has also been suggested that IPNB could be a biliary counterpart of IPMN-P [2, 3]. This hypothesis is supported by the presence of simultaneous intraductal tumors of both the bile duct and pancreas, which are extremely rare. To the best of our knowledge, there have been five reports of patients with simultaneous IPNB of the proximal bile duct (i.e., intrahepatic and perihilar IPNB) and IPMN-P of the pancreas (Table 1) [4–8]. Herein, we would like to present a case of intraductal papillary neoplasm extensively involved along intrahepatic bile duct, extrahepatic bile duct, and pancreas.

## 2. Case Presentation

A 53-year-old man had been experiencing obstructive jaundice and significant weight loss for 1 month. He had been healthy until the jaundice occurred and was not taking a medication on a regular basis. No history of parasitic infestation was noted. Physical examination revealed no obvious finding except for slightly icteric sclera. Abnormal indicators of initial laboratory tests included total bilirubin at 2.3 mg/dL (0.3–1.5), direct bilirubin at 1.6 mg/dL (0–0.5), aspartate aminotransferase (AST)/alanine aminotransferase (ALT) at 122/131 IU/L (<36 IU/L), alkaline phosphatase (ALP) at 464 IU/L (42–121), and CA19-9 at 838.6 (0–37) IU/L.

**Figure 1:**
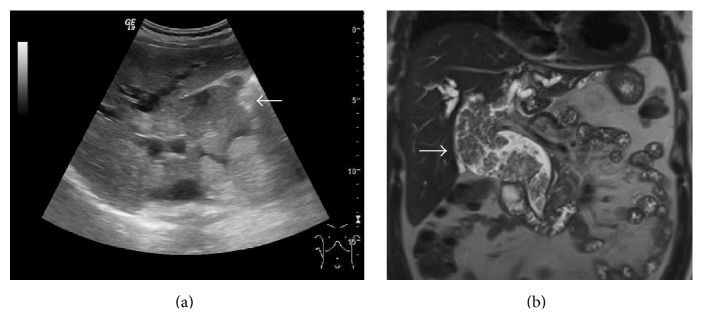
Preoperative imaging. (a) An ultrasonography and (b) MRCP revealed extensive papillary tumor in LHD, CHD, and CBD (arrow). LHD: left hepatic duct, CHD: common hepatic duct, and CBD: common bile duct.

The imaging, including ultrasound and magnetic resonance cholangiopancreatography (MRCP), demonstrated extensive intraductal tumors in the pancreas and biliary system, including the left hepatic duct, the anterior branch of right hepatic duct, the common bile duct down to the distal part, and the entire length of pancreatic duct (Figure 1). Neither lymph node nor distant organ metastasis was detected. Therefore, clinical staging was T2N0M0. Given the patient's preferences, good performance status, and limitation of disease in the bile duct and pancreas, we planned to perform surgery.

The patient underwent right percutaneous transhepatic biliary drainage (PTBD) using an 8.5 Fr. self-retaining catheter. Ten days later, left portal vein embolization (PVE) was performed. A follow-up CT scan was conducted to assure adequacy of the liver remnant.

**Figure 2:**
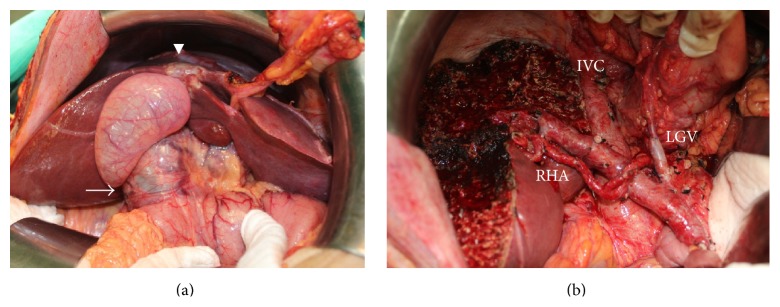
Intraoperative findings. (a) Significant dilatation of extrahepatic bile duct (arrow) and mass-forming lesion at segment 4 (arrow head). (b) Operative field after removal of specimen. IVC: inferior vena cava, LGV: left gastric vein, and RHA: right hepatic artery.

One month later, the operation was carried out. Regarding intraoperative findings, apart from the lesions depicted on preoperative imaging, there was a 2 cm mass-forming lesion at segment 4 of the liver (Figure 2(a)). We decided to perform left trisectionectomy, pyloric-preserving pancreaticoduodenectomy, and total pancreatectomy (Figure 2(b)). The postoperative course was uneventful, except for diabetes mellitus requiring insulin administration.

**Figure 3:**
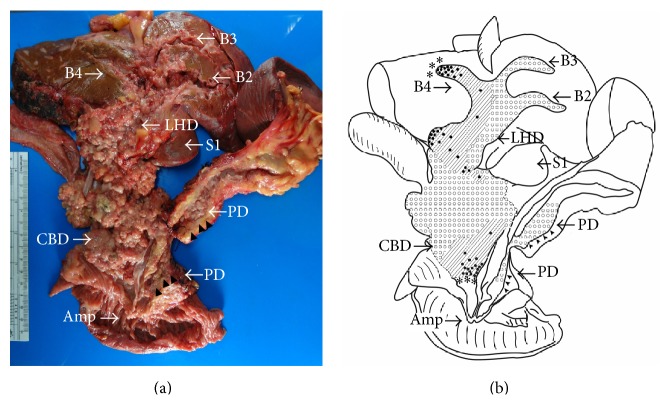
The surgical specimen and a schematic illustration of histopathology grading. (a) The surgical specimen of combined left trisectionectomy, pancreaticoduodenectomy, and total pancreatectomy. The picture shows the opening along extrahepatic bile duct and cut surfaces of liver and pancreas that reveals numerous diffuse soft pink papillary growths occupying the whole extrahepatic bile duct (LHD, RHD, CHD, and CBD) and left intrahepatic segment ducts (B2–B4). The pancreatic head was accidently displaced; therefore, its pancreatic duct (PD) has been labeled by the use of arrow heads. The main PD was diffuse dilation with intraductal lesions prominent at the proximal part, and generalized atrophy of the pancreas parenchyma was noted. No lesion was seen in the gallbladder. (b) A schematic illustration of the surgical specimen histopathological grading showing areas with low-grade (white-dotted area), high-grade (shaded area), carcinoma in situ (black dot), and invasive carcinoma areas located beneath the schema (asterisk). Amp: ampulla of Vater, B2: bile duct of segment 2, B3: bile duct of segment 3, B4: bile duct of segment 4, CBD: common bile duct, CHD: common hepatic duct, LHD: left hepatic duct, PD: pancreatic duct, RHD: right hepatic duct, and S1: segment 1 of liver.

**Figure 4:**
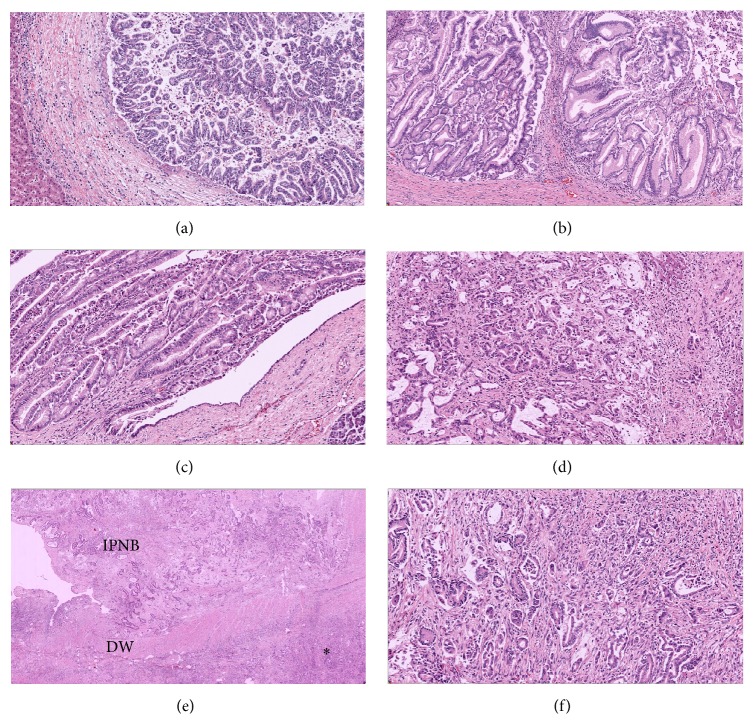
Micrographs. (a–c) Intraductal papillary lesions: (a) intrahepatic bile duct: IPNB with carcinoma* in situ*; (b) common bile duct: low-grade IPNB; and (c) main pancreatic duct: low-grade IPMN. (d–f) Invasive lesions: (d) mucin-producing tubular adenocarcinoma from invasive IPNB lesion at S4 of liver; (e, f) invasive carcinoma from distal common bile duct; (e) invading carcinoma at the duodenal wall (DW) from the intraductal lesion of the distal common bile duct (IPNB), invasive tumor at duodenal wall (*∗*), highlighted in (f) showing moderately differentiated tubular adenocarcinoma. Original magnification: (a–c) ×100, (d, f) ×200, and (e) ×40.

The surgical specimens consisted of the liver (segments 1–5 and 8), gallbladder, extrahepatic bile duct, duodenum, entire pancreas, and spleen. Examination of specimens disclosed numerous intraductal papillary lesions present almost throughout the entire specimen (Figure 3). The histopathology revealed intraductal papillary neoplasm with gastric phenotype of bile duct and pancreas (Figure 4). The IPNB was diffusely located in the dilated intrahepatic bile ducts of B2, B3, B4, and the extrahepatic bile duct, while the majority of the lesion was low-grade intraepithelium neoplasm (Figures 3(b) and 4(b)). High-grade lesions were noted on the left hepatic duct, medial segment duct, and B4 as well as the distal extrahepatic bile duct (Figure 3(b)). The invasive foci were found at B4 and distal bile duct (Figures 3(b) and 4(d)–4(f)), the invading tumor forming a 2 cm subcapsular mass at S4A (Figure 4(d)), and an infiltrative lesion located at the wall of the duodenum (Figures 4(e) and 4(f)), respectively. Metastasis was found in lymph node along common hepatic artery (station 8). In the pancreas, the main pancreatic duct was dilatation, pronounced mostly at the distal part. Low-grade intraepithelium neoplasm (IPMN) was presented in the pancreatic head as intraductal soft pink growths plus multiple indurated nodular lesions (Figures 3 and 4(c)). The epithelium of the distal duct was denuded and the pancreas parenchyma presented atrophic changes. No high-grade lesions or invasive foci were seen in the pancreas.

The patient received Gemcitabine-Cisplatin chemotherapy due to lymph node involvement in the pathological result. A CT scan was performed 5 months after the operation, showing no tumor recurrence.

After the 4th cycle of chemotherapy, the patient developed cholangitis and was admitted at a provincial hospital. During admission, a CT scan was performed revealing multiple peritoneal metastases. In addition to the advancement of the disease, the patient's performance status was also poor. Therefore, the patient received only supportive treatment and eventually passed away. The overall survival time after surgery was 8 months.

## 3. Discussion

We have described a case of extensive involvement of intraductal neoplasm of the bile duct (both extrahepatic and intrahepatic) and the pancreas with a poor outcome despite being aggressively managed by radical surgery and adjuvant chemotherapy.

Since 2001, intraductal papillary neoplasm of the bile duct has been described using the term “intraductal papillary neoplasia in the liver” [9]. Before this term was used, it had been called various names (e.g., biliary papillomatosis, mucin-producing cholangiocarcinoma, and biliary intraductal papillary mucinous neoplasm) [10]. According to the latest classification of bile duct tumors, IPNB is considered to be preinvasive lesions of intraductal growth-type of intrahepatic cholangiocarcinoma and papillary type of extrahepatic cholangiocarcinoma [11]. The natural history of IPNB is usually indolent. It is a slow-growing tumor with lower potential for malignancy than conventional cholangiocarcinoma [11], as it has adenoma-carcinoma sequence in pathogenesis. However, it can develop to macroinvasive lesion, given the poor prognosis, with a one-year survival of 56% [12]. The treatment of IPNB is usually the same as that of cholangiocarcinoma, with the goal being the achievement of R0 resection (no invasive lesion left in patient's body) [13].

IPNB and IPMN-P have been reported as sharing many characteristics. Both of them are composed of fibrovascular cores with neoplastic epithelia and mucin productions have usually been observed [2, 11]. IPNB has been included in the concept “biliary disease of pancreatic counterpart” [14]. This concept is supported by the existence of simultaneous cases of IPNB and IPMN-P [7]. The cause of the association of these diseases remains unclear. From the embryological perspective, both the bile duct and pancreatic duct developed from the same primordium. This might represent “field cancerization,” which is consistent with the multiplicity of the intraductal tumor.

Interestingly, however, the similarities between IPNB and IPMN were limited to proximal-type IPNB (i.e., intrahepatic and perihilar IPNB). It was observed that IPNB of the extrahepatic bile duct, particularly common bile duct, had the features that differed from IPMN-P. They usually were of high grade with invasion [11].

To the best of the authors' knowledge, there have been five reported cases of patients with simultaneous IPNB and IPMN-P. In all of these cases, the biliary involvements were limited to the intrahepatic and perihilar bile duct, which had characteristics similar to IPMN-P and usually progressed slowly. We have presented the first case of extensive intraductal neoplasm involving the extrahepatic bile duct, intrahepatic bile duct, and entire length of the pancreas with a poor outcome, even after being treated aggressively with radical surgery and adjuvant chemotherapy. The cause of the association between IPNB and IPMN-P in our patient was believed to be the effect of field cancerization, rather than the metastatic process, due to area coexistence with varying degrees of dysplasia and invasive lesions. Regarding the treatment of this patient, the decision to perform curative-intent surgery was difficult. There was evidence that curative hepatectomy with R0 resection results in a better survival rate of IPNB patients [15]. To archive R0 resection in this case, extensive surgical resections were required, including left trisectionectomy, pancreaticoduodenectomy, and total pancreatectomy, which carried a high risk of morbidity. Moreover, there was a chance of residual multicentric-dysplastic epithelium in the remnant liver. In this case, the R0 resection was archived without any major morbidity. However, compared to previous reported cases, this patient had a short survival period. This might represent the aggressiveness of IPNB involving the extrahepatic bile duct.

## 4. Conclusion

This case supports the belief that intraductal papillary neoplasm of the bile duct (IPNB) and intraductal papillary mucinous neoplasm of the pancreas (IPMN-P) represent the different locations of a single disease. It also demonstrates the differences in nature according to location of biliary involvement.

## Figures and Tables

**Table 1:** Reported cases of simultaneous intraductal papillary neoplasm of the bile duct and pancreas.

Author	Reported year	Patient	Location of tumor	Treatment	Follow-up	Status
Joo et al. [4]	2000	Male 60 years	LIHD, PD	Surgery	10 months	Alive No recurrence

Ishida et al. [5]	2002	Male 67 years	Segment 1 Uncinate process	Surgery	14 months	Alive No recurrence

Yamaguchi et al. [6]	2005	Male 69 years	LIHD, PD	Surgery	8 months	Alive No recurrence

Zalinski et al. [7]	2007	Female 65 years	LHD, RPHD, PD	Surgery	NA	NA

Valente et al. [8]	2012	Female 76 years	RHD + PD	CCRT CMT (GEM)	36 months	Alive

Present study	2016	Male 53 years	LIHD, LHD, RAHD, CHD, CBD, PD	Surgery Adjuvant CMT	8 months	Dead

CBD: common bile duct, CCRT: concurrent chemoradiation therapy, CHD: common hepatic duct, CMT: chemotherapy, GEM: gemcitabine, IHD: intrahepatic duct, LHD: left hepatic duct, LIHD: left intrahepatic duct, NA: not available, PD: pancreatic duct, and RHD: right hepatic duct.

## List of Abbreviations

CBD: Common bile duct
CHD: Common hepatic duct
CT: Computed tomography
LGV: Left gastric vein
IHD: Intrahepatic duct
IPMN-P: Intraductal papillary mucinous neoplasm of pancreas
IPNB: Intraductal papillary neoplasm of the bile duct
LHD: Left hepatic duct
RAHD: Right anterior hepatic duct
RHA: Right hepatic artery
RHD: Right hepatic duct.
